# Machine learning and integrative analysis identify the common pathogenesis of azoospermia complicated with COVID-19

**DOI:** 10.3389/fimmu.2023.1114870

**Published:** 2023-05-22

**Authors:** Jiarong He, Yuanqiao Zhao, Zhixian Zhou, Mingming Zhang

**Affiliations:** ^1^ Department of Neurosurgery, The Second Xiangya Hospital, Central South University, Changsha, Hunan, PR, China; ^2^ Department of Urology, The Second Xiangya Hospital, Central South University, Changsha, Hunan, PR, China; ^3^ Department of Obstetrics and Gynecology, The Second Xiangya Hospital, Central South University, Changsha, Hunan, PR, China

**Keywords:** azoospermia, COVID-19, single-cell sequencing, machine learning, WGCNA

## Abstract

**Background:**

Although more recent evidence has indicated COVID-19 is prone to azoospermia, the common molecular mechanism of its occurrence remains to be elucidated. The aim of the present study is to further investigate the mechanism of this complication.

**Methods:**

To discover the common differentially expressed genes (DEGs) and pathways of azoospermia and COVID-19, integrated weighted co-expression network (WGCNA), multiple machine learning analyses, and single-cell RNA-sequencing (scRNA-seq) were performed.

**Results:**

Therefore, we screened two key network modules in the obstructive azoospermia (OA) and non-obstructive azoospermia (NOA) samples. The differentially expressed genes were mainly related to the immune system and infectious virus diseases. We then used multiple machine learning methods to detect biomarkers that differentiated OA from NOA. Enrichment analysis showed that azoospermia patients and COVID-19 patients shared a common IL-17 signaling pathway. In addition, GLO1, GPR135, DYNLL2, and EPB41L3 were identified as significant hub genes in these two diseases. Screening of two different molecular subtypes revealed that azoospermia-related genes were associated with clinicopathological characteristics of age, hospital-free-days, ventilator-free-days, charlson score, and d-dimer of patients with COVID-19 (P < 0.05). Finally, we used the Xsum method to predict potential drugs and single-cell sequencing data to further characterize whether azoospermia-related genes could validate the biological patterns of impaired spermatogenesis in cryptozoospermia patients.

**Conclusion:**

Our study performs a comprehensive and integrated bioinformatics analysis of azoospermia and COVID-19. These hub genes and common pathways may provide new insights for further mechanism research.

## Introduction

Infertility affects 10-15% of couples worldwide, and nearly half of these are due to male factors. Despite the high prevalence of male infertility, approximately 70% of patients do not receive a timely clinical diagnosis (1). This knowledge gap prevents clinicians from counseling infertile men about causal treatment and transmission of infertility to offspring. The latter aspect is of particular concern, as low sperm counts and male infertility may be associated with an additive risk for cardiovascular disease, cancer predisposition, and even premature death (2). Viral genomes have been reported in the semen of people infected with Ebola and Zika viruses, which have not previously been identified as sexual transmission (3). The transmembrane serine protease 2 (TMPRSS2) and angiotensin-converting enzyme (ACE) were highly expressed in testicular germ and somatic cells, suggesting that SARS-CoV-2 may be at work in the gonads (4). Rastrelli et al. showed the development of hypogonadotropic hypogonadism and infertility in patients with active COVID-19 cases, demonstrating impaired adult Leydig cell function, although whether an impairment is associated with viral localization in the testis remains unclear (5). Li et al. pointed out that the semen of 6 samples tested positive for COVID-19, with 4 of these patients during the acute phase of infection and 2 at two and three days after clinical recovery, respectively (6). More recently, Gacci et al. demonstrated that sexually active young men in the age range of 30 to 45 years recovering from COVID-19 are at substantial risk of developing oligo-crypto-azoospermia (7). The occurrence of several virus strains has been described in the male reproductive system, particularly the testis. The mumps and HIV virus can directly damage testicular structures, resulting in male infertility (8, 9). Abnormal spermatogenesis has also been described in hepatitis B virus, hepatitis C virus, or herpes simplex virus infections (9).

However, little information about the relationship between azoospermia and COVID-19 has been reported. Therefore, further studies are warranted to explore the potential biomarkers associated with azoospermia and the possible pathomechanisms and common therapeutic targets between azoospermia and COVID-19. We performed impaired spermatogenesis WGCNA analysis, differential expression analysis, and machine learning using public databases. WGCNA is a method widely used in molecular biology to explore patterns of gene interactions among multiple samples (10). It can be used to discover highly co-expression gene sets networks, as well as possible biomarker candidates or therapeutic targets based on the interconnection of gene sets and association with clinical features (11). Additional single-cell sequencing was also performed to confirm our findings. scRNA-seq is a breakthrough approach that allows the clustering of cells to explore the variances of gene expression across all groups and differences in the cell cycle (12, 13). In this context, we used support vector machine-recursive feature elimination (SVM-RFE) and least absolute shrinkage and selection operator (LASSO) classification models to discover features that might distinguish impaired spermatogenesis from OA samples. Random forest (RF) and logistic regression (LR) analyses were used to verify the accuracy of the model. In addition, CIBERSORT was utilized to compare the immune cell infiltration between NOA and OA tissues, as well as COVID-19 and COVID-19-ICU samples. All the samples in COVID-19 cohorts were classified into two discrete groups, based on the four azoospermia-related genes. Moreover, we investigated the relationship between tissue-infiltrating immune cells and diagnostic markers to better understand the cellular and molecular immunological processes involved in the development of impaired spermatogenesis. The highly dynamic process of spermatogenesis is regulated by subtle interactions between the somatic environment and the germline. Moreover, bulk RNA-seq and scRNA-seq analyses of impaired human testicular tissue allowed us to identify multiple distinct spermatogonia states based on transcriptional profiles.

## Materials and methods

### Datasets

**Figure 1:**
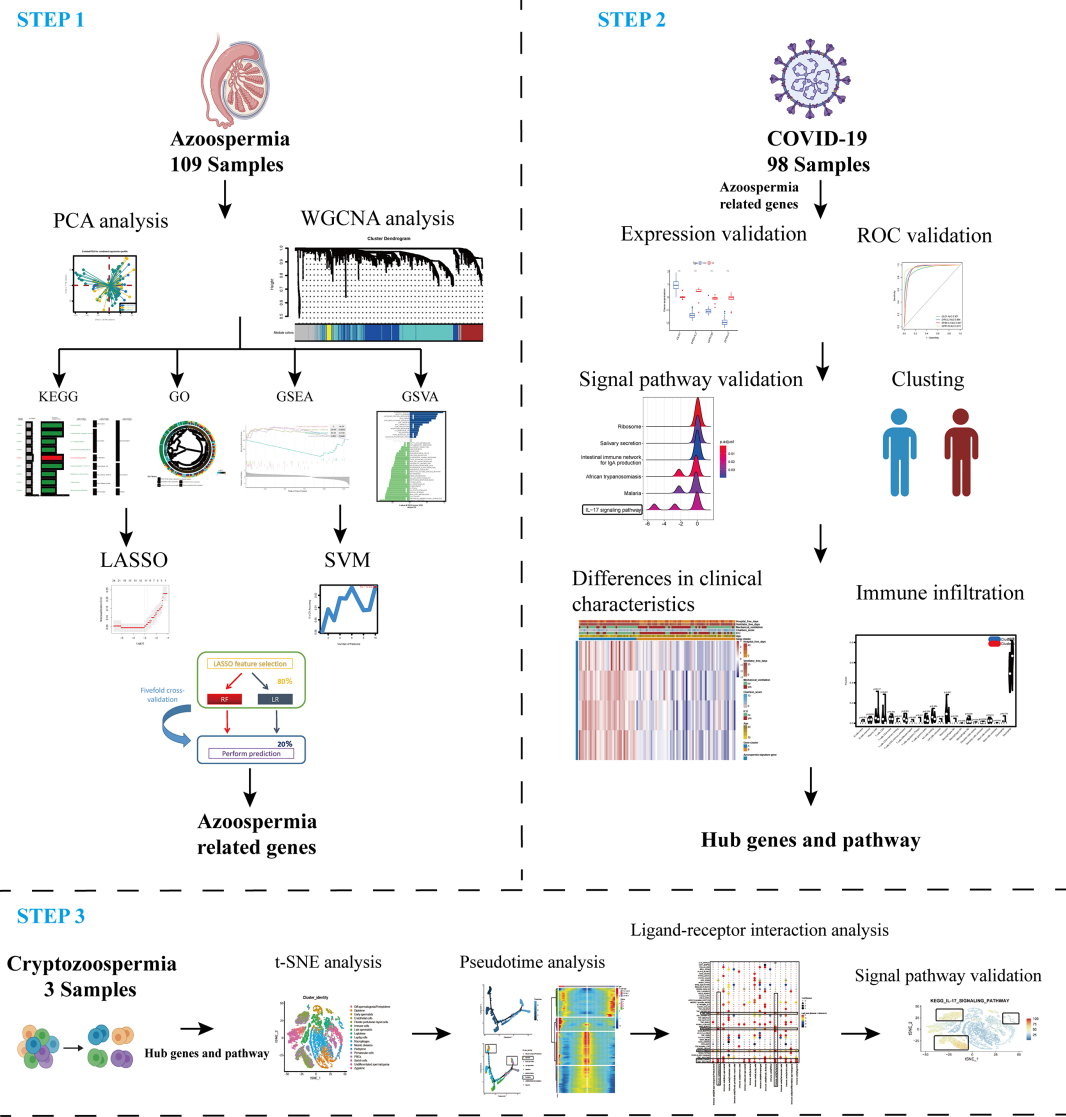
Schematic representation of the bioinformatic analysis process in our present study. WGCNA, machine learning, and scRNA-sequencing data were used to analyze and screen hub targets in azoospermia and COVID-19.

A flow chart that illustrates our research steps can be presented in **Figure 1**. Three azoospermia RNA chip datasets (GSE145467, GSE45885, and GSE9210), one COVID-19 RNA chip dataset (GSE157103), and one cryptozoospermia single-cell RNA-sequencing dataset (GSE153947) were downloaded from the NCBI GEO database (http://www.ncbi.nlm.nih.gov/geo) (14–17). After standardizing and excluding samples without complete information, individual genes were further annotated by respective platforms in the GSE145467 (10 NOA samples and 10 OA samples), GSE45885 (27 NOA samples and 4 OA samples), GSE9210 (47 NOA samples and 11 OA samples). As described previously, we transformed the expression profiles of GEO datasets as TPMs, which was the same as the microarray results (18). The “ComBat” package was used to reduce the influence of batch effects from non-biological and technical biases among the different datasets (19). The heterogeneity of the datasets before and after batch effect removal was examined by the “PCA” package. **Table 1** shows the clinical data of 3 patients with obstructive azoospermia and 3 patients with cryptospermia for single-cell analysis, and **Table 2** lists the clinical data of patients with COVID-19. All the datasets were available from the literature, and the ethics statements of these literature confirmed that all patients provided written informed consent.

**Table 1:** Clinical data of patients with obstructive azoospermia and cryptospermia for single-cell analysis.

Sample	OA 1	OA 2	OA 3	Crypto 1	Crypto 2	Crypto 3
**Age**	31	33	55	39	25	36
**Ejaculate volume (ml)**	0.5	1.1	1.7	1.5	2.1	1.4
**Total sperm count (millions)**	0	0	0	<0.1	<0.1	<0.1
**Sperm concentration (million/ml)**	0	0	0	<0.1	<0.1	<0.1
**FSH (u/l)**	2	3.3	8.2	8.5	19.9	11.6
**LH (u/l)**	2.1	2	7.1	2.5	5.7	4.6
**Total testosterone (nmol/l)**	16.5	25.1	28.3	18.7	13.2	18.1
**SHBG (nmol/l)**	49	69	66	38	28	26
**Free testosterone (pmol/l)**	268	336	404	368	296	441
**Prolactin (mu/l)**	158	127	227	176	146	174
**Estradiol (pmol/l)**	61	104	201	71	63	84
**DHT (nmol/l)**	1.68	1.75	1.08	42.4	1.01	1.34
**Testicular volume (ml)**	17.5	21	21.5	8.5	14	7.5
**Bergmann-kliesch score**	9	8	8	1	0	4
**Tubules with elongated spermatids**	90	76	81	12	0	38
**Tubules with round spermatids**	2	3	6	3	0	4
**Tubules with spermatocytes**	5	17	10	3	0	26
**Tubules with spermatogonia**	1	0	1	2	0	1
**Tubules sertoli cell only**	0	0	0	80	95	26
**Tubular shadows**	1	3	2	0	5	5
**Retrieval of sperm from the tese**	Yes	Yes	Yes	Yes	Yes	Yes
**Karyotype**	46, XY	46, XY	NA	46, XY	46, XY	46, XY

**Table 2:** Clinical data of patients with COVID-19 for analysis of differential genes and common pathways.

Characteristics	COVID cohort	Normal cohort
	(n=62)	(n=12)
Age		
≤65	35(56.4)	7(58.3)
>65	26(41.9)	5(41.7)
NA	1(1.7)	0
Gender		
Male	62(100)	12(100)
ICU		
Yes	33(53.2)	8(66.7)
No	29(46.8)	4(33.3)
Charlson score		
≤5	53(85.4)	8(66.7)
>5	9(14.6)	4(33.3)
Mechanical ventilation		
Yes	29(46.8)	5(41.7)
No	33(53.2)	7(58.3)
Ventilator free days		
≤15	20(32.2)	2(16.7)
>15	42(67.8)	10(83.3)
Hospital free days		
≤30	36(58.1)	3(25)
>30	26(41.9)	9(75)

### WGCNA-derived modular signature

The “WGCNA” R package to find modules of strongly associated genes with the Weighted Gene Coexpression Network. Primarily, soft-thresholding powers were determined using the pickSoftThreshold function. Subsequently, establish a WGCNA network. The minimum number of genes in a module is 46, soft-thresholded power of 12, and a dendrogram cut height of 0.3. Succeeding, WGCNA, which clusters genes into modules based on correlations between gene expression patterns (10). To distinguish modules, each module with a unique color identifier and gray representation with the remaining mismatched genes. According to the correlation coefficient of MTR analysis and the visualized expression trend of each module. Picked two modules (MEturquoise and MEblue), on account of exhibiting the highest positive/negative correlation and revealing gradually increasing or decreasing expression trends.

### Screening and verification of diagnostic markers

We performed the LASSO, and SVM-RFE to screen for novel azoospermia biomarkers. The “glmnet” and “e1071” packages in R were utilized to implement the LASSO and SVM, respectively (20, 21). The three candidate genes were then figured out. This research used the RF method in the “randomForest” package to validate biomarkers using five-fold cross validation. Furthermore, LR and RF were used as the cross validation set for providing an in-depth assessment of the effectiveness of selected biomarkers (22, 23).

### DEGs identification and ROC analysis

The “limma” R Package was employed to investigate the differences between the COVID-19 and normal sample subgroups, COVID-ICU and COVID-Non-ICU subgroups. After applying a filter (|logFC| > 2 and adjusted P < 0.05), we obtained DEGs and displayed them by heatmap graph respectively. The selected genes were used to identify biomarkers with high sensitivity and specificity for azoospermia and COVID-19 diagnosis. The receiver operator characteristic curves were plotted and the area under the curve (AUC) was calculated separately to evaluate the performance of each gene using the R packages “pROC”. AUC > 0.75 indicated that the gene had a good diagnosis effect (24).

### Signature selection methods

The upregulated genes and downregulated genes were handled separately with the XSum method. Then, the change values were sums of the reference/compound signatures relative to increased query/disease genes (sum-up) and decreased query/disease genes (sum-down). In brief, XSum is defined as the following equation: XSum=sum-up−sum-down (25).

### Functional and pathway enrichment

We explore the biological processes associated with genes in these key modules. The Gene Ontology (GO) and Kyoto Encyclopedia of Genes and Genomes (KEGG) analyses based on DEGs were conducted by the “clusterProfiler” R package. Gene Set Variation Analysis (GSVA), an unsupervised analysis method, is non-parametric. It evaluated the enrichment of different signaling pathways of genes in the modules with different samples (26). Gene set variation analysis was performed using the “limma” and “GSVA” packages. To identify signaling pathways differentially activated in the modules. We performed Gene Set Enrichment Analysis (GSEA) analysis with adjusted P < 0.05 using the “clusterProfiler” R package (27).

### Evaluation of immune cell infiltration

To evaluate the abundance of immune infiltrates, We uploaded the gene expression matrix data to CIBERSORT (https://cibersort.stanford.edu/) and obtained the immune cell infiltration matrix (28). Then, we used the “corrplot” package to draw a correlation heatmap to visualize the correlation of 22 types of infiltrating immune cells. ssGSEA was performed by the GSVA R package to analyze the infiltration of 24 immune cells of four screened genes (GLO1, DYNLL2, EPB41L3, GPR135) (26).

### Single-cell quality control, normalization, and cell type annotation

The “SingleR” R package was utilized to annotate scRNA-seq data automatically. Cells expressing 200 to 2500 genes were identified, while those expressing<10% of mitochondrial genes were retained. After 3000 hypervariable genes were identified and analyzed, the number of main components was calculated to annotate cell clusters that were then visualized using the “tSNE” algorithm. The same dimensional reduction and clustering approaches were applied to the spermatogonia subgroup of the crypt and normal datasets. To enable an attempt to verify the cluster identities without bias, we calculated the top marker genes in each cluster using the “FindMarkers” algorithm and tested with the MAST method for positive cluster marker genes that were least expressed in 30% (P < 0.001) (29). The marker genes were used with well-known cell type-specific markers from the previous literature to subsequently assign their module identities (30–33).

### Single-cell signature explorer

The Single-Cell Signature Scorer was compiled for GNU Linux and Microsoft^©^Windows™ 64 bits, and can be compiled for any platform using cross-compilation by Go. https://academic.oup.com/nar/article/47/21/e133/5531181.

### Cell-cell communication analysis

CellphoneDB software (Version 2.1.2) was used for cell-cell communication analysis using the “statistical analysis” method. The threshold for ligands and receptors was required to be expressed by at least 50% of all cells, and the maximum number of iterations was set at 10,000. Interactions were considered significant at P < 0.05.

### Pseudotime analysis

Single-cell pseudotime trajectories were generated using the Monocle2 algorithm to reflect cell-state transitions (34). The “differentialGeneTest” package was applied to calculate the DEGs over the Pseudo-time among all cluster cell transitions. “DDRTree” was used for dimensionality reduction and visualization, and the “plot cell trajectory” function was performed to visualize the differentiation trajectory of cells.

### Statistical analysis

All statistical analyses and graphical visualization were generated using the R software, (https://www.r-project.org, v4.0.2). Correlations were calculated as Spearman’s rank-order correlation coefficient unless stated otherwise, and pairwise comparisons were tested using Wilcoxon rank-sum and Kruskal-Wallis tests. pROC package was used to determine the ROC curves and AUC values. All data points represent individual biological replicates, not technical replicates. A two‐tailed P‐value of <0.05 was considered statistically significant unless indicated otherwise.

## Results

### Data preprocessing and construction of the weighted gene co-expression network

**Figure 2:**
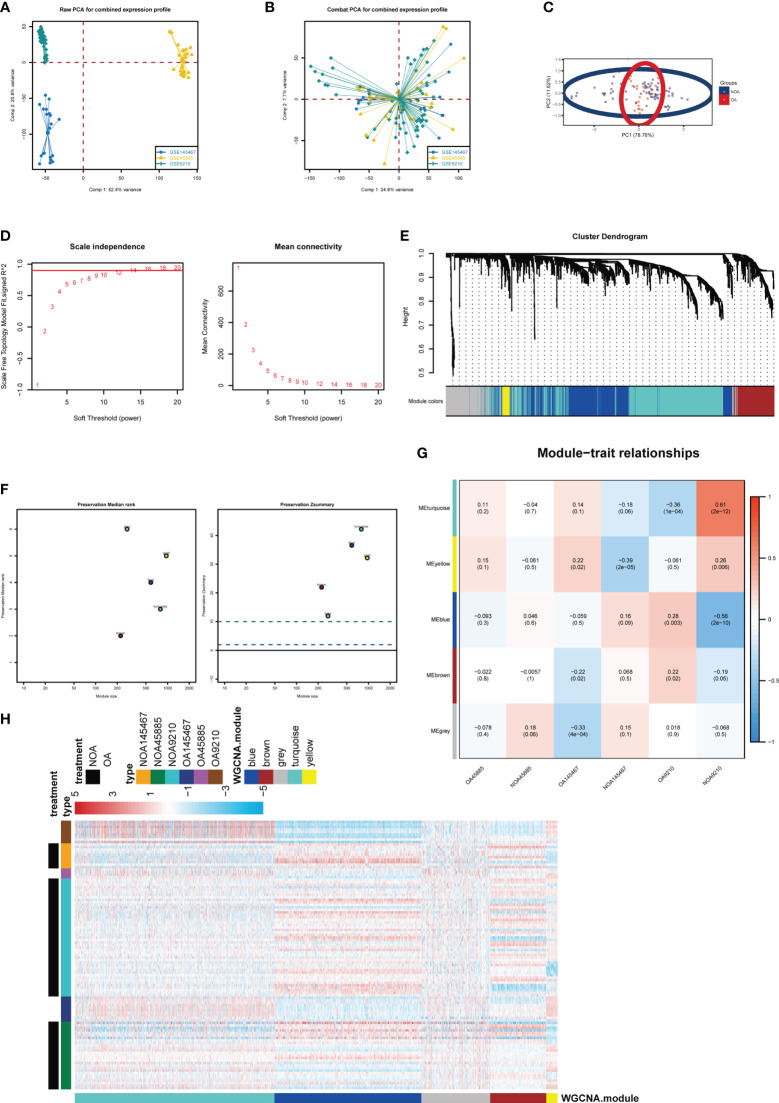
Key modules identified by WGCNA. **(A)** PCA plot before batch correction of gene expression profile in azoospermia dataset. **(B)** PCA result after z-score normalization. **(C)** Principal component analysis of different groups. **(D)** On the left panel, the x‐axis shows the soft power threshold and the y‐axis presents the scale-free topology. On the right panel, the x-axis indicates the soft power threshold and the y-axis indicates mean connectivity. **(E)** The cluster dendrogram of the WGCNA. **(F)** The preservation median rank and z-summary scores of each module. **(G)** Module-region associations. Each column represents a sample and each row represents a consensus module eigengene. Each sample contains the corresponding correlation coefficient and P-value. Each panel was color-coded by correlation according to the accompanying legend. **(H)** Heatmap representation for clustering of differentially expressed genes.

To explore and validate the potential regulatory pattern in testicular samples from azoospermia patients, we combined the microarray analysis of tissues obtained from azoospermia patients and performed intra-omics normalization batch correction (**Figures 2A, B**). The PCA result showed that the GSE9210 group, GSE145467 group, and GSE45885 group had good reproducibility and reproducibility (**Figure 2C**). Here, the soft-thresholding power parameter was set to 12 to fulfill the scale-free topology model, in which the red line (R2) was used to judge how well the model fits the scale freeness (**Figure 2D**). Then, we observed 5 network modules, whose connectivity was shown in a WGCNA cluster dendrogram (**Figure 2E**). While the “grey” module contained the unassigned genes identified as not co-expressed. The individual genes corresponding to each module classification are listed in **Table S1**. By comparing the OA datasets with the NOA datasets, the composite summary preservation statistic, a statistic that determined whether the genes in a reference module can be explained by another process in the test network, was visualized. Modules ‘turquoise’ and ‘blue’ were found to be the most stable across samples (**Figure 2F**). The highest correlation in the module-trait relationship was found between the turquoise module (r = 0.61, P=2e-12) and the blue module (r = -0.56, P=2e-10; **Figure 2G**), which were selected for the subsequent analyses. The gene expression profile for each module in individuals of each group was shown (**Figure 2H**).

### Functional analysis of critical module genes

**Figure 3:**
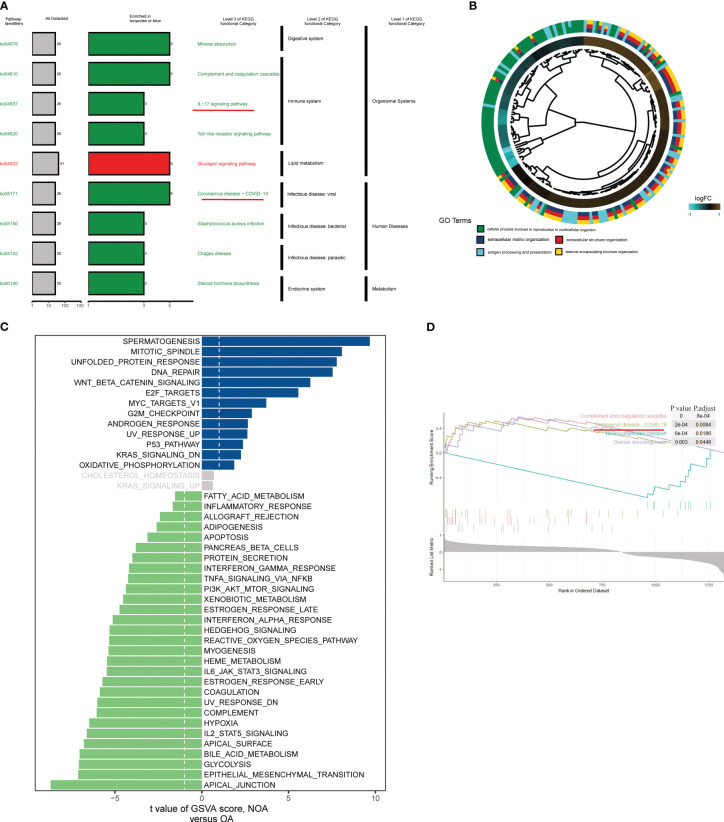
Biological processes and pathways in the azoospermia modules were significantly associated with COVID-19. **(A)** KO salient functional categories that were significantly enriched in the turquoise module were shown in green, while those that are significantly enriched in the blue module were shown in red. **(B)** Circos plot showing relationships between GO terms and the module genes. Log2 fold changes (FC) of gene expression are represented by colored squares. **(C)** Differences between NOA subject and control subject GSVA pathway scores were determined (n= 84 and 25 samples from 109 patients, respectively). The t-value obtained by a linear mixed model. **(D)** GSEA of representative KEGG signaling pathways.

To further investigate the pathways involved in all differential KO functional categories, KEGG pathway enrichment analysis was performed separately for the KO functional categories enriched in turquoise and blue modules (**Figure 3A**). The “Complement and coagulation cascades,” “IL-17 signaling pathway,” and “Coronavirus disease − COVID−19” pathways were enriched in the turquoise module. By contrast, the “Glucagon signaling pathway” was significantly enriched in the blue module. Cross-examination of GO terms revealed that a substantial number of DEGs associated with the extracellular process were enriched for biological functions such as antigen processing and presentation (**Figure 3B**). A direct comparison of NOA versus OA performed by GSVA analysis identified “SPERMATOGENESIS” and “APICAL_JUNCTION” targets as the top enriched hallmarks (**Figure 3C**). In addition, gene set enrichment analysis also revealed significant coronavirus disease pathway changes in key modules of the azoospermia (**Figure 3D**).

### Feature selection to identify azoospermia-related genes

**Figure 4:**
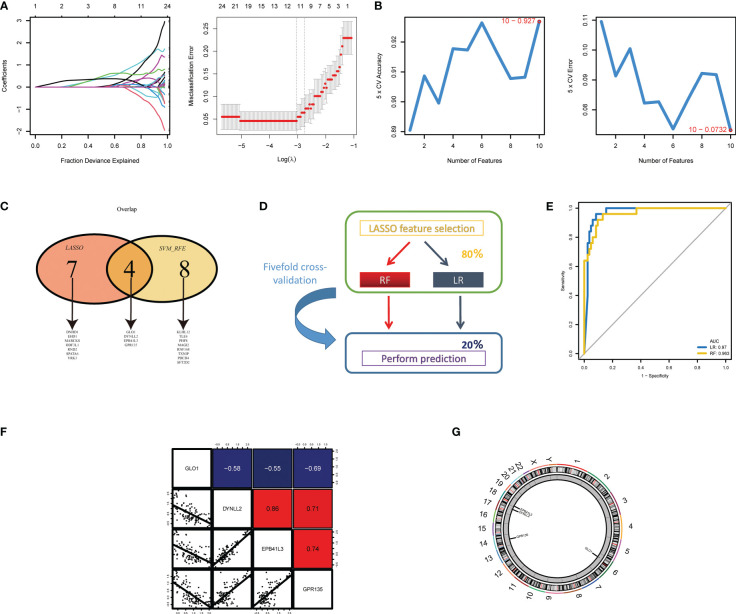
Screening and validation of the diagnostic indicators for azoospermia. **(A)** LASSO and **(B)** SVM-RFE algorithms were selected for feature selection in the discovery cohort. **(C)** Venn diagram showing the overlapping markers in the two comparisons indicated. **(D)** Schematic representation of predictors construction and feature selection by 5-fold internal cross validation across the test set. **(E)** The ROC curves of the two models are based on their AUC. **(F)** Correlations among GLO1, DYNLL2, EPB41L3, and GPR135 expression levels in azoospermia tissues. **(G)** The alterations of CNV locations in azoospermia-related genes on 23 chromosomes.

Next, two different algorithms were used to select the most significant biomarkers for classifying NOA and OA patients. First, we performed lasso logistic regression models to identify 11 potential biomarkers from the turquoise and blue module genes (**Figure 4A**). Second, the SVM-RFE algorithm was performed and a set of 12 critical biomarkers was selected (**Figure 4B**; **Table S2**). A total of 19 azoospermia-related genes were identified by combining the biomarkers selected by LASSO and SVM-RFE, of which 4 genes were selected simultaneously by both algorithms (**Figure 4C**). In the test set, two established machine-learning methods were applied to 5-fold cross-validation and their performance was evaluated on the basis of AUC scores (**Figure 4D**). Strikingly, we found that the expression profiles of four genes could accurately classify patients with azoospermia. Logistic regression and random forest, achieved a high AUC of 0.970 and 0.963 on the independent test set, respectively (**Figure 4E**). Then, correlation analysis was performed to investigate the association between these four genes and azoospermia. The expression levels of GPR135 in azoospermia tissues positively correlated with the levels of “DYNLL2” and “EPB41L3” and negatively correlated with GLO1 expression (**Figure 4F**). Also, the location of the selected biomarkers on chromosomes was downloaded from the Ref-seq database annotation, as shown in **Figure 4G**.

### Validation of DEGs in the COVID-19 dataset

**Figure 5:**
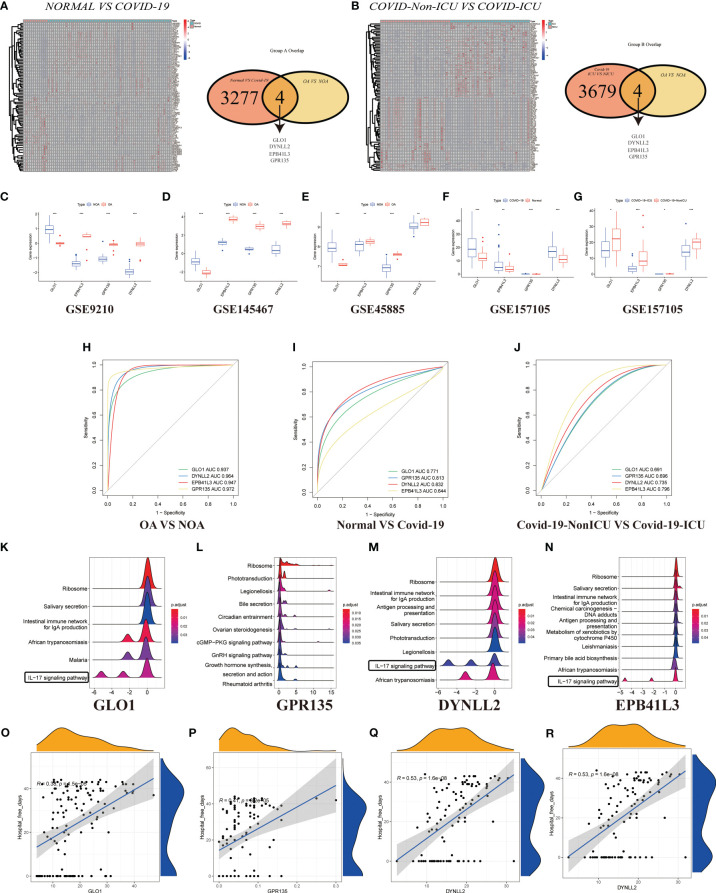
Validation of DEGs in GSE157103. **(A)** Heatmap clustering of candidate genes with markedly expression patterns in COVID-19 compared to normal samples. Green denotes COVID-19 samples, whereas red denotes normal samples. Adjusted P-value < 0.05 and |log2FC| > 2 were considered significant. Venn diagram showing the number of overlapping markers. P values were calculated with the use of Wald tests. **(B)** Heatmap clustering of candidate genes with substantial expression patterns in COVID-ICU compared to COVID-Non-ICU samples. Expression levels of four DEGs in azoospermia **(C–E)** and COVID-19 datasets **(F, G)**. P values were set as: *P < 0.05; **P < 0.01; ***P < 0.001. ROC plots of the specifically co-expressed hub genes in azoospermia **(H)**, COVID-19 **(I)**, and COVID-19-ICU **(J)** patients. The enriched item in GSVA analysis (**K**, GLO1; **L**, GPR135; **M**, DYNLL2; **N**, EPB41L3). **(O–R)**. Correlation analysis between four azoospermia-related genes and hospital free days.

In GSE157103, we identified 4501 differentially expressed genes between hospitalized patients with COVID-19 and those without COVID-19 (62 and 12, respectively) (**Figure 5A**), including 3120 up-regulated genes and 159 down-regulated genes (**Table S3**). Additionally, a total of 62 samples were analyzed, including 33 from patients with COVID-19 in ICU and 29 were obtained from patients with COVID-19 who were not in ICU (**Figure 5B**; **Table S4**). Then generated gene lists were combined to retain only the overlapping genes. The expression levels of three DEGs were detected in GSE9210, GSE145467, GSE45885, and GSE157105 datasets. Comparing the expression levels of corresponding genes in NOA tissues and OA tissues, it can be concluded that GLO1 expression was up-regulated, while GPR135, DYNLL2, and EPB41L3 gene expression was down-regulated (**Figures 5C–E**). In the COVID-19 database, the expression of four genes was up-regulated in the treatment group (**Figures 5F, G**, P < 0.05). ROC curves were used to evaluate the expression of the above four genes among the azoospermia and COVID-19 samples. The AUC combines both sensitivity and specificity to authenticate the predictive validity of diagnostic coding. Of these, GPR135 exhibited the highest diagnostic performance in azoospermia samples (AUC = 0.972). The diagnostic performances of the other genes were measured as follows: GLO1 (AUC = 0.937), DYNLL2 (AUC = 0.964), and EPB41L3 (AUC = 0.947) (**Figure 5H**). These above-mentioned genes could be considered candidate diagnostic biomarkers for azoospermia. The diagnostic efficacy of these azoospermia-related genes was also verified in the COVID-19 dataset. DYNLL2 had high accuracy for the diagnosis of COVID-19 (AUC = 0.832), and EPB41L3 had certain accuracy for the diagnosis of COVID-19-ICU (AUC = 0.796) (**Figures 5I, J**). According to these four azoospermia-related genes, GSVA enrichment analyses were performed **(** **Figures 5K–N** **)**. GSVA pathway analysis showed that the differentially expressed genes in COVID-19 patients were also mainly involved in the IL-17 signaling pathway, which was consistent with the **Figure 3A** key module enrichment analysis results of azoospermia patients (**Figures 5K, M, N**). Meanwhile, we also found that the azoospermia-related genes were positively correlated with the hospital free days of COVID-19 patients (P < 0.05), suggesting that they may also be associated with the prognosis of COVID-19 **(** **Figures 5O–R** **)**.

### The characteristics of azoospermia-related subtypes in COVID-19

**Figure 6:**
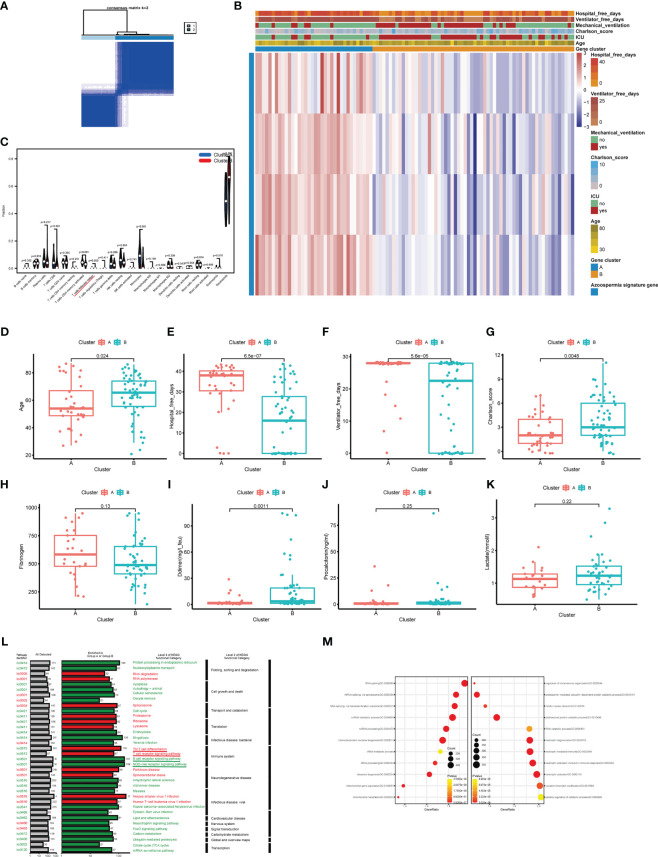
Clinicopathological and immune characteristics of COVID-19 subtypes based on azoospermia-related genes. **(A)** The consensus clustering matrix of 62 samples in the GSE157103 cohort (k = 2). **(B)** Heatmap of the clinicopathologic features classified by these DEGs. **(C)** The infiltrating scores of 22 immune cells in the two clusters. Associations between the two clusters and clinicopathological characteristics **(D)**. Age, **(E)** Hospital free days, **(F)** Ventilator free days, **(G)** Charlson score, **(H)** Fibrinogen, **(I)** D-dimer, **(J)** Procalcitonin, **(K)** Lactated. **(L)** KO salient functional categories that were significantly enriched in COVID-Non-ICU were shown in green (Group A), while those that were significantly enriched in COVID-ICU were shown in red (Group B). **(M)** The right shows the GO enrichment analysis of DEGs between COVID-19 and normal samples, while the left shows the GO enrichment analysis of DEGs between COVID-ICU and COVID-Non-ICU samples.

To explore the relationship between the expression of the azoospermia-related genes and COVID-19 subtypes, we performed the consensus cluster analysis to classify patients with COVID-19. By applying the K-means clustering algorithm, the intra-group correlation was the highest and the inter-group correlation was lowest when k = 2. The results indicated that the 62 patients with COVID-19 were divided into two clusters (cluster A, n = 25; cluster B, n =37) based on the 4 DEGs (**Figure 6A**). The gene expression profile (GEP) and corresponding clinicopathological parameters, including the degree of hospital free days, ventilator free days, mechanical ventilation, charlson score, ICU, and age, were presented in a heatmap (**Figure 6B**). To investigate the effect of azoospermia-associated genes on the immune microenvironment of COVID-19, we evaluated the infiltrating immune levels of every COVID-19 sample between the two subtypes using the CIBERSORT algorithm (**Table S5**). Our study indicated that cluster B showed higher infiltration fractions of T cells follicular helper and neutrophils (**Figure 6C**). Screening of two different molecular subtypes revealed that azoospermia-related genes were associated with clinicopathological characteristics of COVID-19 (**Figures 6D–K**). We observed a higher number of hospital free days and ventilator free days among patients with cluster A. However, the age and d-dimer of cluster B were higher than those of cluster A (P < 0.05). Information of the KO functional categories enriched in Group A (Normal vs COVID-19) or Group B (COVID-ICU vs COVID-Non-ICU) was also separately conducted using KEGG pathway enrichment. The “Cell growth and death” and “Immune system” pathway including Apoptosis, B cell receptor signaling pathway, and NOD-like receptor signaling pathway, exhibited higher relative abundance in the enrichment of COVID-Non-ICU compared to that of COVID-ICU. In contrast, the “T cell receptor signaling” and “Th17 cell differentiation” pathways were significantly enriched in COVID-ICU (**Figure 6L**). To determine the biological functional categories between the two groups, we performed GO enrichment analysis to identify the potential function diversities. Remarkably, we detected that plenty of RNA-related pathways were enriched in COVID-ICU and neutrophil-associated pathways in COVID-Non-ICU (**Figure 6M**).

### Immunological infiltration analysis in the azoospermia dataset

**Figure 7:**
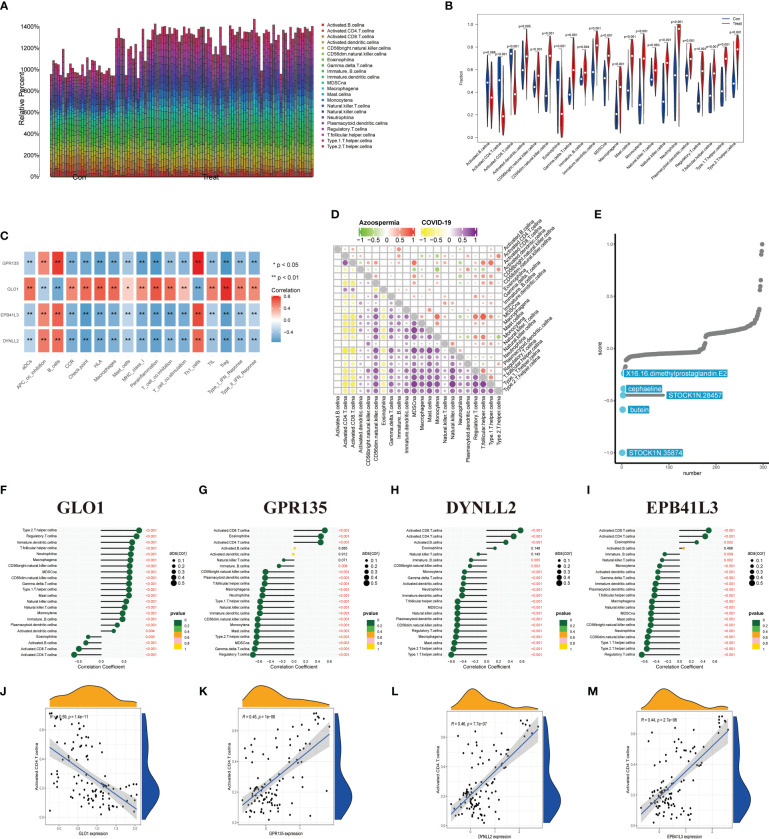
Correlation between infiltrating immune cells and diagnostic markers. **(A)** Relative percent of 22 immune cells in the control and treat group. **(B)** The infiltrating scores of 22 immune cells in the control and treatment group. **(C)** Correlation analysis of different immune cell scores estimated by CIBERSORT. **(D)** Microenvironmental immune cell profiling of azoospermia and COVID-19. **(E)** Results of overall best-hit practice approach-based computational predictions as query signature. Top-ranked five compounds with the highest reversal potency scores were illustrated in the panel. **(F–I)** Correlation between infiltrating immune cells and GLO1, GPR135, DYNLL2, and EPB41L3. The size of the dots indicates the degree of correlation between genes and immune cells and is proportional to the correlation strength. The color panel of the dots indicates the range of P-value. **(J–M)** Correlations between four DEGs and activated CD4 T cells.

We then investigated the degree of immune infiltration in OA and NOA patients and found that immune cells were generally enriched in the treatment group compared with the control group (**Figure 7A**), and T helper cells, which are closely related to IL-17 signaling, were enriched in the treatment group (**Figure 7B**, P < 0.05). To further investigate the potential relationship between the above differential genes and the differential immunoscores, we performed a correlation analysis of the CIBERSORT enrichment scores of these genes. The results indicated that the score of “GLO1” was significantly negative correlated with the CIBERSORT scores of “Th1 cells” (R > 0, P < 0.01), and the immune expression pattern of GLO1 in azoospermia patients was opposite to that of the other three genes (**Figure 7C**). On the other hand, we used the ssGSEA method to calculate the infiltrating immune cell types in patients with azoospermia and COVID-19. The total ssGSEA score (the sum of absolute scores across 22 leukocyte components) was remarkably higher in the samples with COVID-19 than those in the azoospermia, including activated CD4 T cells, activated CD8 T cells, macrophages, and T helper cells (**Figure 7D**; **Table S6**). Using the compromising parameter (topN=500) and the optimal method (XSum), the pairwise similarity scores of all compounds were obtained (lower scores correspond to higher reversal potency and better therapeutic potential for application). Notably, Cmap analysis found STOCK1N. 35874, the enrichment score was -1.0, which could be used as a potential therapeutic target for NOA (**Figure 7E**). According to the results of immune correlation analysis between OA and NOA samples, GLO1 showed a positive correlation with T helper cells (P < 0.001), but it displayed a negative correlation with activated CD4 T cells (P < 0.001) (**Figure 7F**). While the remaining three genes had opposite associations with activated CD4 T cells and T helper cells as GLO1 (**Figures 7G–I**). The correlation analysis between the four DEGs and activated CD4 T cells was shown in **Figures 7J–M**.

### Single-cell dimension reduction clustering and cellular changes in somatic and germ cell compartments

**Figure 8:**
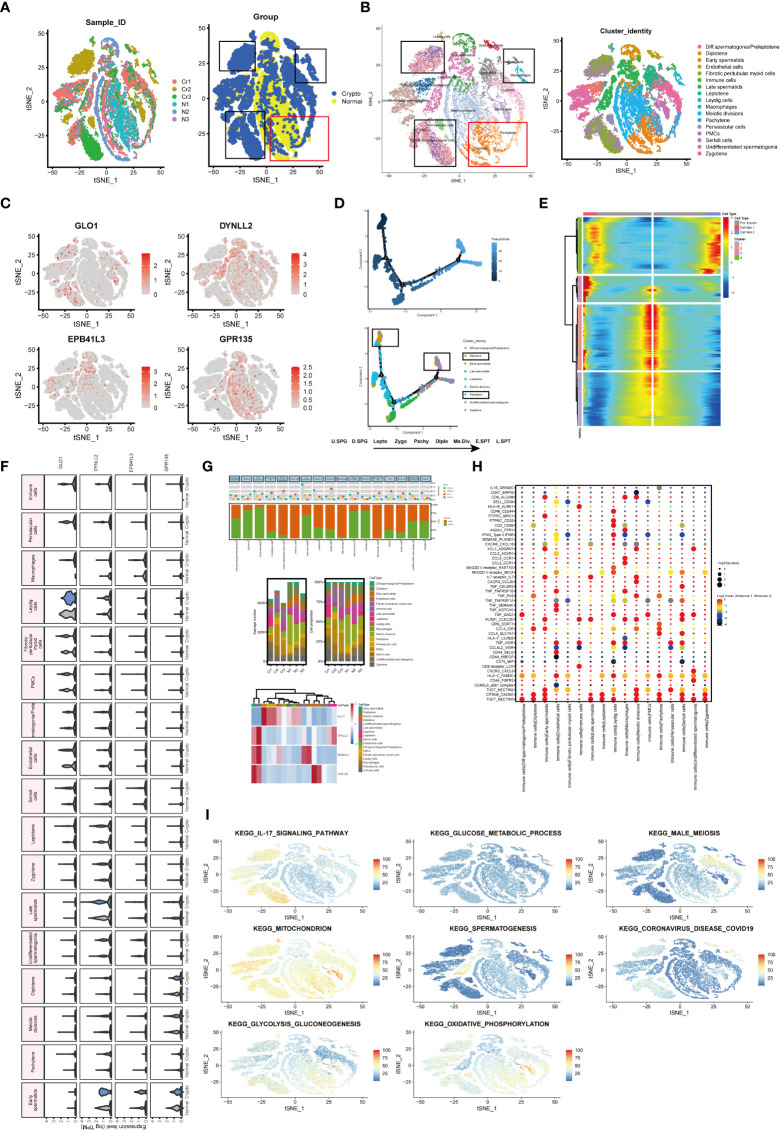
Exploration of transcriptional and cellular alterations in crypto and normal testicular tissues. **(A)** t-distributed stochastic neighbor embedding (t-SNE) plot of the integrated crypto and normal datasets. **(B)** scRNA-seq data from the crypto and normal samples. Each cell type denotes a different color. **(C)** Feature plots of four azoospermia-related genes. **(D)** Pseudotime analysis discovered the developmental trajectories of crypto samples. **(E)** Heatmap showing azoospermia-related genes involved in germ cell differentiation (Branch point1). **(F)** Violin plots visualizing the expression levels of candidate marker genes for each cell type. **(G)** Three-layered structure of potential cell marker genes for each cell cluster and sample. Mean expression values of known lineage markers (top panel); Relative proportion of subsets and average cell number for each sample (middle panel); Relative expression profiles of four marker genes correlated with each cell subpopulation (bottom panel). Mean expression levels were scaled by mean centering and converted to a log 2 scale. **(H)** Summary of ligand-receptor interaction analysis between the immune cells and the rest of the cell types in the crypto tissues. The P-value was indicated by the size of the corresponding circle. The color gradient scale indicates the degree of interaction. **(I)** The distribution of eight biological processes in GSE153947.

To determine whether azoospermia-related genes and IL-17 signaling pathway were compatible in cryptozoospermia. The molecular changes were characterized in depth by scRNA-seq analysis of crypto and normal testicular biopsies (n = 3 each) to assess the similarities and differences in the two sets of groups. After quality control filtering, data from 15,532 and 13,134 cells, respectively, were ultimately included in the analyses of normal and cryptozoospermia samples (**Table S7**). To verify the accuracy of cell annotation, we checked the expression of acknowledged cell-specific markers within each cell cluster that was annotated directly by the “SingleR” R package (**Figures 8A, B**). The relative expressions of four azoospermia-related genes were shown in **Figure 8C**. GLO1 expression was mainly concentrated in the crypto group, while the remaining three genes were mainly expressed in the control group, which is consistent with the immune infiltration of NOA disease. The development of cryptozoospermia cells is a dynamic process. The use of the Monocle2 algorithm to speculate about the possible developmental trajectories of cryptozoospermia cells revealed that the trajectory began with undifferentiated spermatogonia cells and ended with late spermatids cells (**Figure 8D**). We found deviations in the developmental trajectories of pachytene cells and diplotene cells located at branch point 1. In addition, we explored the dynamic change of azoospermia-related genes during germ cell development (Branch point 1, **Figure 8E**).

Next, we sought to detect azoospermia-associated changes of gene expression in germ and somatic cells. The SCENIC analysis discovered the evenly distributed germ cell populations along with similarly expressed azoospermia related genes in normal and cryptozoospermia samples, while significant differences were observed between immune cells and perivascular cells (**Figure 8F**). We observed most germ cell clusters belong to normal tissue, of which five types have been assigned to known cell types, consisting of pachytene, meiotic divisions, late spermatids, early spermatids, and diplotene cells. On the contrary, somatic cell clusters were specifically enriched in perivascular cells, macrophages, immune cells, fibrotic peritubular myoid cells, and endothelial cells present in crypto samples (**Figure 8G**). The relative proportion of subsets and average cell number for each sample were shown in the middle panel. The panel of heatmap revealing the RNA expression of four marker genes was found in **Figure 8G**. To investigate the interaction between spermatogonia and their microenvironment, CellphoneDB was used. In the crypto dataset, we identified significant ligand-receptor interactions. Considering the azoospermia-related genes were not differentially expressed in diplotene and pachytene but differentially expressed in immune cells, we explored the ligand-receptor pair relationship between immune cells and them. As shown in **Figure 8H**, TNF, CCL3, HLA-C, CRTAM, and TIGIT secreted by immune cells interact with receptors expressed on both diplotene and pachytene cells. These ligand-receptor pairs might be involved in the immune pathway and spermatogenesis. However, no ligand-receptor pair directly related to IL-17 signaling was found. Furthermore, we investigated the relationship between the cell types and biological processes and found that the IL-17 signaling pathway and coronavirus disease enriched in a subgroup of crypto, while the male meiosis and spermatogenesis process narrowly distributed in the crypto subgroup (**Figure 8I**).

## Discussion

In this study, we identified four genes, GLO1, GPR135, DYNLL2, and EPB41L3 that were strongly associated with azoospermia and COVID-19. We performed this analysis using weighted co-expression networks, machine learning, and differential expression analysis of existing azoospermia datasets. ROC curve analysis then revealed that these genes were capable of accurately diagnosing azoospermia/COVID-19 and our findings show that their expression may be related to T helper cells. In addition, azoospermia-related genes shared IL-17 signaling pathway in both diseases by enrichment analysis were also observed. Then, two distinct molecular subtypes of COVID-19 were identified based on four azoospermia-related genes. Patients with cluster A had fewer T cells follicular helper and worse clinicopathological features than patients with cluster B. Finally, we used a novel Xsum method to predict drug therapeutic targets based on the expression of four key genes in azoospermia patients. To uncover additional biomarkers and novel molecular pathways associated with azoospermia progression, we analyzed GEO bulk data (GSE9210, GSE145467, and GSE45885) and GSE153947 (used as a single-cell validation dataset). These four genes were then screened using two machine learning algorithms with unique properties (lasso regression and support vector machine), and further validated using logistic regression and random forest algorithms. GLO1 is the enzyme that catalyzes the glutathione-dependent detoxification of the compound methylglyoxal (MG), thereby protecting against cellular injury and necrosis. It is commonly overexpressed in numerous human malignancies as a newly identified survival strategy by providing an additional safeguard through the enhancement of GLO1 detoxification (35). GLO1 inhibitors have been investigated for their effects on dicarbonyl stress in various pathologies, including atherosclerosis (36), diabetes and its vascular complications (37), osteoporosis (38), anxiety-linked behavior (39), and age-related decline in heart function (40). Zhang et al. reported that follicle-stimulating hormone and total testosterone-dependent upregulation of GLO1 maintains porcine Sertoli cell viability by controlling the argpyrimidine- and hydroimidazolone-mediated NF-κB pathway (41). On the other hand, Motawa et al. suggested that MG may functionally inactivate the COVID-19 proteome and that GLO1 inhibitors may possess antiviral activity against COVID-19 (36). The results of our study indicated that the mRNA expression of GLO1 was higher in NOA samples compared to OA samples. Moreover, the expression level of GLO1 was also found to be higher in COVID-19 patients than in normal individuals. G-protein-coupled receptors (GPCRs) are key mediators of signal transduction pathways and attractive targets for pharmacological therapeutics. GPR135, a GPCR, has received limited research attention, and its involvement in azoospermia and COVID-19 remains unclear. Previous studies have shown that GPR135 activators can suppress tumor activation and are associated with affective disorders (42). Our findings indicate that GPR135 has high diagnostic accuracy in azoospermia samples and a positive correlation with the length of hospital-free days in COVID-19 patients. However, further research is required to elucidate its specific mechanism of action.

Of the remaining two genes, members of the LC8 family of dynein light chain isoforms (DYNLL1 and DYNLL2) are ubiquitous, highly conserved eukaryotic homodimer proteins in addition to dynein and myosin 5a motor proteins, with numerous (still incomplete) proteins involved in diverse cellular processes (43). DYNLL2 has been identified as a novel prognostic biomarker for ischemic stroke and osteosarcoma, but its role in azoospermia and COVID-19 has not been revealed (44, 45). Our study demonstrated a positive correlation between DYNLL2 and T helper cells, as well as an association with the IL-17 signaling pathway in azoospermia and COVID-19. Additionally, DYNLL2 exhibited a high diagnostic value as determined by the ROC in both conditions. DAL-1, also known as EPB41L3, plays a critical role in cytoskeleton-related processes and interacts with various protein molecules *via* its FERM, SAB, and CT domains. Loss of DAL-1 expression, often caused by abnormal DNA methylation and/or LOH, is commonly observed in cancer (46). Nevertheless, additional studies are necessary to investigate whether EPB41L3/DAL-1 can serve as a reliable diagnostic biomarker with high sensitivity and specificity for azoospermia and COVID-19. EPB41L3 confers supportive and resilient to animal cell membranes and facilitates the assembly of several multimeric protein complexes. It also plays important roles in tumor suppression, and cell proliferation regulation, and is highly enriched in the testis, suggesting that it has a previously undiscovered function in reproduction (47). Our findings revealed that the mRNA expression of EPB41L3 was lower in NOA samples when compared to OA samples. Conversely, in patients with COVID-19, the expression level of EPB41L3 was higher than that observed in normal individuals. More recent studies seem to support an effect of COVID-19 infection on male sex steroid hormones, namely an increase in plasma LH levels and a significant decrease in FSH and testosterone levels (48). Of these, one out of four patients with healed COVID-19 (11/43, 25.5%) were diagnosed with oligo-, crypto-, or azoospermia, a percentage significantly exceeding that reported in the general population (approximately 1% for azoospermia (49); 3% for oligozoospermia (50)). Surprisingly, all azoospermia cases reported unimpaired prior fertility status (one had three children, two had two children, and five had one child). Most importantly, semen concentration was associated with febrile episodes during and after meiosis, with mean reductions of 32.6% and 35%, respectively (51). Particularly, one-quarter of the sample who have recovered from COVID-19 exhibit signs of male genital tract inflammation and oligo-, crypto-, and azoospermia, which strictly correlate with disease severity. This would not only provide valuable information about the biology of human reproduction but may also reveal the possible mechanisms behind the observed association between male infertility and COVID-19 (4). Four azoospermia-related genes emerged in our study that has not been reported to be related to the IL-17 signaling pathway so far in the literature, and further investigations are needed.

In this work, we performed a detailed study of human male germ cell developmental defects using scRNA-seq to examine whether azoospermia-related genes have a relationship with cryptozoospermia. These observations led us to decipher target genes and mechanisms for understanding the etiology of infertility. In addition, the receptor-ligand interactions operating the interplay between germ cells and their microenvironment were explored. Interestingly, the same state was found in both the crypto and normal groups. Although the total amount of spermatogonia remained the same, the states of the two groups presented different proportions. Based on the scRNA-seq data, we identified some biological progress that was enriched in specific cells. Compared with the normal group, the cryptozoospermia group (Fibrotic peritubular myoid cells, Perivascular cells, PMCs, Immune cells, and Macrophages) was mainly gathered in the process of IL-17 signaling pathway and coronavirus infection, while the normal group (Diplotene and Pachytene) was mainly concentrated in the process of male meiosis and spermatogenesis. This phenomenon was consistent with the previous study (7). These studies may pave the way for understanding the link between COVID-19 and male infertility.

However, this study also has some methodological limitations due to the relatively small effect size: (1) The potential biomarkers and pathways found in this research need to be further verified to provide clinical trial evidence for targeted therapy; (2) The accuracy of azoospermia/COVID-19 assessment and prediction could be enhanced by increasing the number of sample size; (3) The analysis of protein expression level of marker genes can provide significant evidence. However, executing validation experiments is problematic due to the lack of appropriate normal testis samples in our laboratory. We intend to collect testis tissue to further understand how the marker genes affect azoospermia in the future. Additionally, incorporating functional studies and experimental validations, such as gene knockout or knockdown experiments, could further elucidate the biological relevance of our identified biomarkers. Future studies will continue to elucidate the underlying mechanism in azoospermia and COVID-19 patients.

## Data availability statement

The original contributions presented in the study are included in the article/**Supplementary Material**. Further inquiries can be directed to the corresponding author.

## Ethics statement

This study was approved by the Academic Committee of the Second Xiangya Hospital of Central South University and all patients were treated following the standardized procedures (No.2021-613). The patients/participants provided their written informed consent to participate in this study.

## Author contributions

JH, YZ, and MZ designed the study and carried out experiments. JH, YZ, and ZZ analyzed the data. JH and YZ wrote the draft of the manuscript. MZ edited the manuscript. All authors contributed to the article and approved the submitted version.
